# Immunosuppressive treatment and the risk of diabetes in rheumatoid arthritis

**DOI:** 10.1371/journal.pone.0210459

**Published:** 2019-01-23

**Authors:** Siri Lillegraven, Jeffrey D. Greenberg, George W. Reed, Katherine Saunders, Jeffrey R. Curtis, Leslie Harrold, Marc C. Hochberg, Dimitrios A. Pappas, Joel M. Kremer, Daniel H. Solomon

**Affiliations:** 1 Diakonhjemmet Hospital, Oslo, Norway; 2 NYU Hospital for Joint Diseases, New York, New York, United States of America; 3 Corrona, LLC, Worcester, Massachusetts, United States of America; 4 UMass Medical School, Worcester, Massachusetts, United States of America; 5 Corrona, LLC., Southborough, Massachusetts, United States of America; 6 University of Alabama at Birmingham, Birmingham, AL, United States of America; 7 University of Massachusetts Medical School, Worcester, Massachusetts, United States of America; 8 University of Maryland School of Medicine, Baltimore, MD, United States of America; 9 Columbia University, College of Physicians and Surgeons, New York, NY, United States of America; 10 Albany Medical College and The Center for Rheumatology, Albany, NY, United States of America; 11 Brigham and Women's Hospital, Boston, Massachusetts, United States of America; VU University Medical Center, NETHERLANDS

## Abstract

**Objective:**

Inflammation and anti-inflammatory treatments might influence the risk of diabetes. The objective of this study was to assess factors associated with incident diabetes in rheumatoid arthritis (RA).

**Methods:**

The study population consisted of RA patients from a multi-center cohort study, Corrona. To assess risk associated with disease modifying antirheumatic drug (DMARD) exposure, we assessed five mutually exclusive DMARD groups. Additionally, we assessed the risk associated with body mass index (BMI, <25, 25–30, >30 kg/m2) and glucocorticoid usage. Incident cases of diabetes were confirmed through adjudication, and Cox regression models were fit to estimate the risk of incident diabetes.

**Results:**

We identified 21,775 DMARD treatment regimens, the mean (SD) age at the index visit was 58 (13) years, disease duration 10 (10) years, and 30% used oral glucocorticoids at the time. Eighty-four incident cases of diabetes were confirmed within the treatment exposure periods. The hazard ratio (HR, 95% confidence interval) for diabetes was significantly reduced in patients receiving TNF inhibitors, HR 0.35 (0.13, 0.91), compared to patients treated with non-biologic DMARDs other than hydroxychloroquine and methotrexate. Hydroxychloroquine, methotrexate and use of other biologic DMARDs had a numerically reduced risk compared to the same group. Patients prescribed ≥7.5 mg of glucocorticoids had a HR of 2.33 (1.68, 3.22) of incident diabetes compared with patients not prescribed oral glucocorticoids. RA patients with a BMI >30 had a HR of 6.27 (2.97, 13.25) compared to patients with BMI ≤25.

**Conclusion:**

DMARDs, glucocorticoids and obesity influenced the risk of incident diabetes in a large cohort of RA patients. Monitoring for the occurrence of diabetes should be part of routine RA management with a focus on specific subgroups.

## Introduction

Rheumatoid arthritis (RA) is a systemic inflammatory disease, characterized by joint pain, loss of function and decreased quality of life [1]. During the last twenty years, the treatment for RA has improved greatly, mainly due to the introduction of biologic disease modifying anti-rheumatic drugs (bDMARDs) and more aggressive treatment strategies, especially in early RA [1–3]. RA patients increasingly receive treatment tailored to their disease activity, co-morbidities and predictors of subsequent joint damage and loss of function.

Diabetes is a growing health problem worldwide. In 2015, 450 million people were estimated to live with diabetes, of which more than 90% of cases are due to type 2 diabetes [4]. Diabetes increases the risk of cardiovascular disease, blindness, kidney failure and lower-limb amputation, and causes substantial morbidity and mortality in affected individuals [4]. RA is associated with abnormalities in the glucose metabolism, mainly insulin resistance, a precursor to type 2 diabetes [5, 6], and data suggest an increased occurrence of diabetes in RA patients, although studies are somewhat conflicting [7–9]. Tumor necrosis factor (TNF)-alpha and IL-6 are involved in the pathogenesis of RA and insulin resistance and diabetes [9]; systemic inflammation drives hyperglycemia [10, 11]. Blockade of interleukin-1 with the drug anakinra has been shown to improve glycemia and beta-cell secretory function in patients with type 2 diabetes [12]. Obesity represents the main risk factor for diabetes; it also correlates with higher RA disease activity, more disability and an increased risk of comorbidities in RA [13, 14].

The association between diabetes and RA raises important questions about whether anti-inflammatory treatment in RA affects the likelihood of diabetes development. In a study conducted in US administrative claims data, TNF inhibitors and hydroxychloroquine were associated with a reduced risk of diabetes when compared to other non-biologic DMARDs (nbDMARDs) [15]. Other studies support that DMARDs, especially hydroxychloroquine, might influence glucose tolerance and diabetes risk in RA [16–19]. Hydroxychloroquine has also shown a beneficial effect on glycosylated hemoglobin (HbA1c) in diabetic RA patients [20]. Glucocorticoid use has been found to be associated with diabetes in RA patients [21], although the reduction of inflammatory activity with glucocorticoid treatment might modulate the relationship between glucocorticoids and diabetes in RA patients [22].

Understanding factors associated with diabetes risk in RA should help guide the clinician in treatment choices, especially in patients with other known risk factors for diabetes. In this study, we assessed if choice of DMARDs and use of glucocorticoids is associated with incident diabetes in RA after adjusting for other important risk factors for diabetes, such as body mass index (BMI). We also study the relationship between obesity and diabetes in RA.

## Material and methods

### Study design

The Consortium of Rheumatology Researchers of North America (Corrona) RA registry is a US multicenter longitudinal observational study [23, 24]. More than 250 centers participate in Corrona, including both academic and non-academic study sites. Corrona participants are assessed at routine clinical encounters, and the data collection includes reports of comorbidities by the patient and physician, clinical assessment, drug utilization recorded at each visit, patient reported outcome measures, and disease activity measures. The study was performed in compliance with the Declaration of Helsinki, and all participants provided a written informed consent. Approvals for practice-level data collection and analyses in Corrona are obtained from local institutional review boards of participating academic sites and central institutional review boards (Western and New England Institutional Review Boards) for private practice sites.

### Exposure variables

The index date for each treatment regimen was the first study visit where the physician reported prescribing the DMARD. The exception was ongoing DMARD use at enrollment into Corrona, which was included in the exposure assessment. DMARD exposure was classified in five mutually exclusive groups predefined in the study protocol (Table 1). **1)** TNF inhibitors, including combinations with any nbDMARD; **2)** other biologic DMARDs (bDMARDs), including combinations with any nbDMARD; **3)** methotrexate, including combinations with nbDMARDs except hydroxychloroquine; **4)** hydroxychloroquine, including combinations with nbDMARDs except methotrexate; and **5)** other nbDMARDs not used in combination with methotrexate, hydroxychloroquine or bDMARDs (leflunomide, cyclosporine, sulfasalazine, azathioprine, minocycline, auranofin, penicillamine). The last group, other nbDMARDs, was chosen as the reference group for the analyses. Treatment regimens with either a combination of biologics or a combination including both methotrexate and hydroxychloroquine were excluded from the analyses. Each subject could not contribute more than four treatment regimens, and treatment regimens were excluded if the patient had a diagnosis of diabetes at the index date of the treatment regimen according to case report forms, or if no follow-up information was available.

**Table 1:** The classification of DMARD regimens applied in the analyses.

*Exposure Groups*	*Inclusion criteria for exposure group*	*Exclusion criteria for exposure group*
TNF inhibitors	Use of etanercept, infliximab, adalimumab, certolizumab, golimumab	Combination with any bDMARDs
Other bDMARDs	Use of abatacept, rituximab, anakinra, tocilizumab	Combination with any bDMARDs
Methotrexate	Use of methotrexate	Combination with any bDMARD or hydroxychloroquine
Hydroxychloroquine	Use of hydroxychloroquine	Combination with any bDMARD or methotrexate
Other nbDMARDs	Use of leflunomide, cyclosporine, sulfasalazine, azathioprine, minocycline, auranofin, penicillamine	Use of any bDMARD, methotrexate or hydroxychloroquine

**Abbreviations**: bDMARD: biologic diseasease modifying anti-rheumatic drug, DMARD: Disease-modifying anti-rheumatic drug, nbDMARDs: non-biologic disease modifying anti-rheumatic drugs, TNF: tumor necrosis factor.

In the analysis of the relationship between glucocorticoid use and diabetes development, glucocorticoid exposure was categorized according to the daily dosage of oral glucocorticoids reported at the index date: none, 1–2.5 mg, 3–7 mg or 7.5 mg or more. BMI was calculated based on height and weight (weight in kilograms / (height in meters x height in meters)).

### Study population

**Fig 1:**
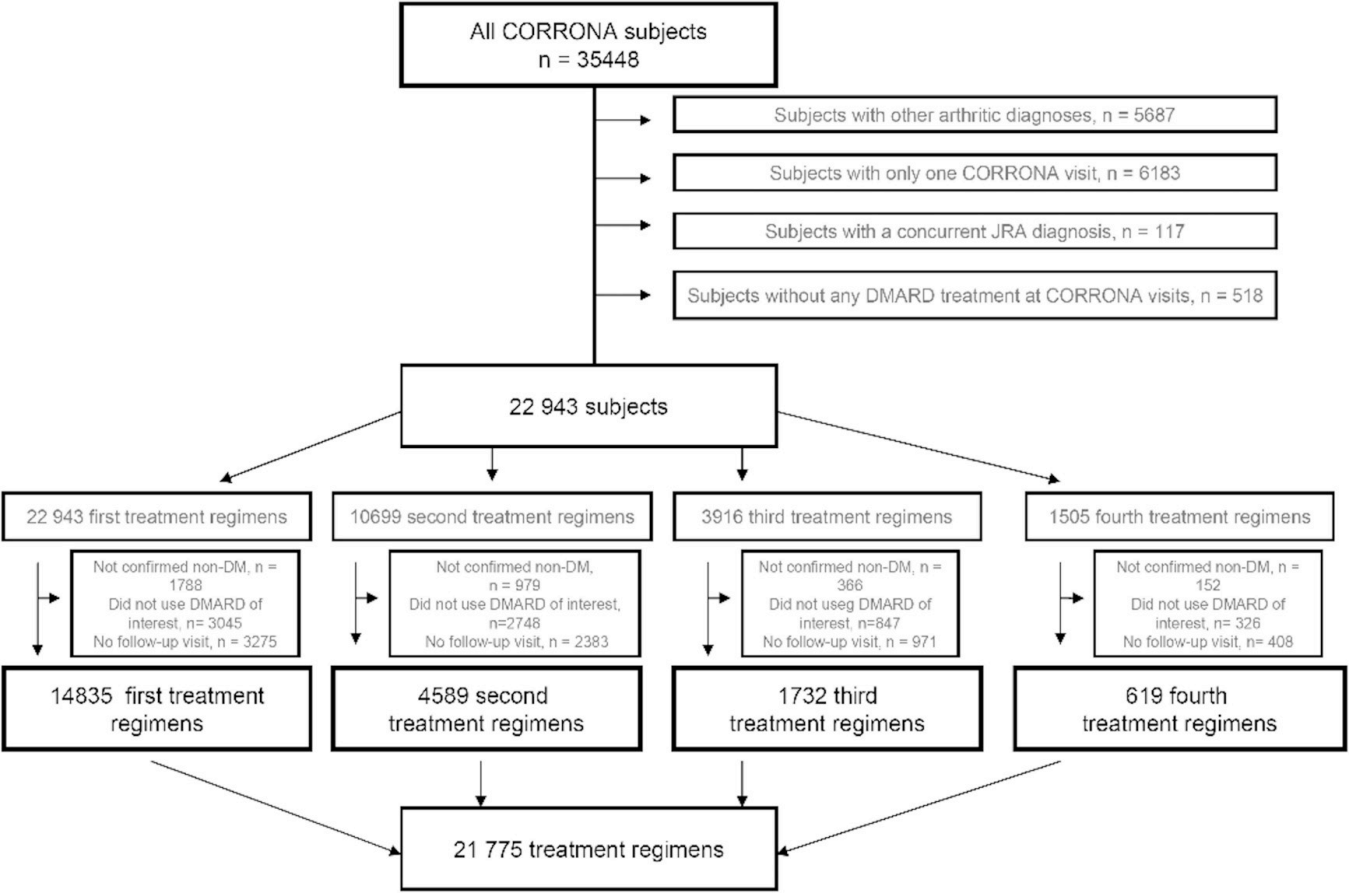
Flow-diagram. Illustration of the selection of treatment regimens for the analyses.

The selection of the study population is outlined in Fig 1. The Corrona dataset included 35448 subjects. We excluded patients with other non-RA forms of arthritis (n = 5687), patients who did not receive DMARDs (n = 518), and patients with only one Corrona visit (n = 6183) or concurrent juvenile idiopathic arthritis (n = 117), which left us with 22943 RA subjects with potential treatment regimens of interest (Fig 1). In these patients, we included treatment regimens with the defined DMARD exposure groups and any follow-up time in patients who did not have a diagnosis of diabetes, which left us with 21,775 treatment regimens of interest.

### Outcome

Incident diabetes represents the main study outcome. The presence of diabetes was registered on the physician questionnaire at each visit, and adjudication was undertaken for all reported incident cases. Adjudication was based on a combination of chart reviews and patient interviews by the individual investigation sites to confirm that the registered diabetes cases were in fact newly diagnosed, and included reported onset of anti-diabetic medication and glycated hemoglobin (HbA1C) if available. Patient-reported diagnosis of diabetes in the case report form was not sufficient to be classified as an incident diabetes case. The case ascertainment questionnaire also asked investigators to confirm the date of diabetes diagnosis. If the dates of diagnosis reported at the Corrona visit and the chart review did not match, the date reported in the chart review was chosen for the analyses. Cases occurred between July 30^th^ 2002 and July 1^st^ 2011.

### Statistical methods

Mean values with standard deviation (SD) and percentages were used to describe characteristics at the index visit of the treatment regimen as appropriate. Person-time, incidence rates (cases per 1,000 person-years) with 95% confidence intervals (CIs) and incidence rate ratios with 95% CIs were calculated for each treatment category, with other nbDMARDs as the reference category for the ratios.

Pair-wise propensity scores were calculated for each DMARD group with other nbDMARDs as the comparator. Propensity score models included Clinical Disease Activity Index (CDAI) [25] pain assessed on an visual analogue scale (VAS), RA disease duration, age, BMI, gender, white vs. non-white race, insurance status, exercise status, subcutaneous nodules, glucocorticoids (dichotomized, no glucocorticoids prescribed vs. glucocorticoids prescribed), physical function assessed by the Modified Health Assessment Questionnaire (MHAQ [26]), history of bDMARD use and history of nbDMARD use. A broad range of variables was included in the propensity model to create a robust model with regards to individual confounding. In sensitivity analyses, history of bDMARD use was left out of the model.

To adjust for differences between DMARD treatment groups, we assessed Cox regression models with adjustment for 1) age and gender 2) age, gender and BMI at onset of treatment and 3) continuous propensity scores from the models described previously. In sensitivity analyses, models adjusted for quintiles of propensity scores and continuous propensity score with additional direct adjustment for BMI were assessed.

The associations of glucocorticoids and BMI to diabetes were assessed in separate Cox regression models. Overweight was defined as a BMI of 25–30 kg/m2, obesity was defined as a BMI of >30 kg/m2. Models were adjusted for the covariates thought to be the most relevant risk factors of diabetes in this setting, namely age, family history of diabetes and DMARD treatment.

Variables with missing values at the index date of the treatment regimen and likely stable throughout the disease course were replaced with information from other visits if available, including gender, race, smoking status, insurance and exercise. Time-varying variables and stable variables still missing after replacement from other visits were imputed by multiple imputation.

Confidence intervals for incidence rates and incidence rate ratios were calculated in Episheet (www.drugepi.org). IBM SPSS Statistics version 20 was used for all other statistical calculations.

## Results

### Patient characteristics

The mean (SD) age at the index visit was 58.2 (13.4) years and the mean disease duration 10.0 (9.8) years (Table 2). The mean score for the disease activity index CDAI was 13.4 (12.4), corresponding to moderate disease activity. Seventy-six percent of the population was women, and 30% had a prescription of oral glucocorticoids at the index visit (Table 2).

**Table 2:** Characteristics at start of treatment regimens for all patients, and each DMARD category separately for Corrona cohort.

*Variables*	*All treatment regimens n = 21 775*	*TNF inhibitors n = 9880*	*Other bDMARDs n = 1756*	*Methotrexate n = 7441*	*Hydroxychloroquine n = 1496*	*Other nbDMARD n = 1202*
Age, mean (SD)	58.2 (13.4)	56.3 (13.2)	57.8 (13.1)	60.5 (13.3)	58.3 (13.7)	60.4 (13.1)
Disease duration, mean (SD)	10.0 (9.8)	10.6 (9.6)	12.4 (9.6)	8.9 (10.0)	8.2 (9.2)	10.9 (10.1)
CDAI, mean (SD)	13.4 (12.4)	13.5 (12.5)	18.2 (13.9)	13.0 (12.2)	9.9 (10.1)	12.5 (11.9)
BMI, mean (SD)	28.7 (6.8)	28.7 (6.8)	29.1 (7.3)	28.7 (6.7)	28.4 (6.8)	28.4 (6.5)
28 swollen joint count, mean (SD)	4.3 (5.5)	4.2 (5.5)	5.4 (5.8)	4.5 (5.8)	2.7 (3.9)	4.1 (5.5)
28 tender joint count, mean (SD)	3.9 (5.7)	4.0 (5.8)	5.7 (6.7)	3.6 (5.4)	2.8 (4.7)	3.4 (5.3)
MHAQ, mean (SD)	0.36 (0.45)	0.36 (0.44)	0.53 (0.50)	0.33 (0.44)	0.30 (0.41)	0.34 (0.47)
Women, n (%)	16643 (76.4)	7670 (77.6)	1426 (81.2)	5458 (73.4)	1219 (81.5)	870 (72.4)
Smokers, n (%)	3516 (16)	1638 (17)	260 (14.9)	1197 (16.1)	220 (14.7)	189 (15.7)
Peroral glucocorticoid use, n (%)	6538 (30)	2706 (27.4)	712 (40.5)	2331 (31.3)	404 (27.0)	385 (32.0)
Insurance, n (%) *None*	340 (1.6)	128 (1.3)	21 (1.2)	167 (2.2)	16 (1.1)	8 (0.7)
*Only medicaid*	494 (2.3)	212 (2.1)	35 (2.0)	179 (2.4)	34 (2.3)	34 (2.8)
*Only medicare*	3255 (14.9)	1231 (12.5)	303 (17.3)	1300 (17.5)	217 (14.5)	204 (17.0)
*Private insurance*	13901 (63.8)	6538 (66.2)	1311 (74.7)	4399 (59.1)	955 (63.8)	698 (58.1)
*Medicare and medicaid*	500 (2.3)	231 (2.3)	45 (2.6)	168 (2.3)	34 (2.3)	22 (1.8)
*Missing*	3285 (15.1)	1540 (15.6)	41 (2.3)	1228 (16.5)	240 (16.0)	236 (19.6)

**Abbreviation:** BMI: Body mass index, CDAI: clinical disease activity index, n: number of patients, SD: standard deviation, DMARD: disease modifying anti-rheumatic drug.

### Incident diabetes

We identified 82 incident cases of diabetes classified to have occurred within the follow-up time defined for the treatment regimens included in the analyses. The diabetes incidence ranged from 1.29 (95% CI 0.43, 3.07) cases per 1,000 person-years in patients receiving hydroxychloroquine to 3.07 (95% CI 1.37, 6.03) cases per 1,000 person-years in patients receiving other nbDMARDs. With other nbDMARDs as the reference, the incidence rate of diabetes was lowest for patients using hydroxychloroquine (incidence rate ratio 0.42 [95% CI 0.12, 1.44]) and TNF inhibitors (incidence rate ratio 0.47 [95% CI 0.21, 1.07]), but these ratios included the null.

### Association between DMARDs and diabetes in multivariate models

In multivariate Cox regression models adjusting for continuous propensity scores, exposure to TNF inhibitors was associated with reduced incidence of diabetes, with a hazard ratio of 0.35 (95% CI 0.13, 0.91, p-value 0.03, Table 3). Reduced hazard ratios were observed for the other DMARD categories, but these findings were not statistically significant **(**Table 3). In sensitivity analyses, similar results were found in Cox regression models adjusted for 1) propensity score quintiles, 2) propensity scores and BMI, 3) propensity scores without history of bDMARD use and 4) bDMARD use and propensity scores without history of bDMARD use.

**Table 3:** Cox regression models assessing the relationship between DMARD exposure and diabetes.

	*Unadjusted models*			*Adjusted for age and gender*			*Adjusted for age*, *gender and BMI*			Propensity score adjusted model*		
	HR	95% CI	P-value	HR	95% CI	P-value	HR	95% CI	P-value	HR	95% CI	P-value
TNF inhibitors vs other nbDMARDs	0.47	0.21, 1.06	0.07	0.50	0.22, 0.13	0.09	0.49	0.21, 1.11	0.09	0.35	0.13, 0.91	0.03
Other bDMARDs vs other nbDMARDs	0.49	0.14, 1.70	0.26	0.49	0.14, 1.78	0.27	0.50	0.14, 1.80	0.29	0.44	0.08, 2.57	0.36
Methotrexate vs other nbDMARDs	0.67	0.30, 1.52	0.34	0.68	0.30, 1.53	0.35	0.64	0.28, 1.46	0.29	0.67	0.44, 1.02	0.34
Hydroxychloroquine vs other nbDMARDs	0.42	0.12, 1.42	0.16	0.42	0.12, 1.46	0.17	0.42	0.12, 1.47	0.18	0.45	0.13, 1.53	0.21

* Propensity score models included CDAI, pain VAS, disease duration, age, BMI, gender, white vs. non-white, insurance, exercise, subcutaneous nodules, glucocorticoids, MHAQ, history of bDMARD use, history of nbDMARD use.

### Influence of glucocorticoids and BMI on the risk of diabetes

The risk of diabetes development increased with increasing doses of glucocorticoids. Patients using ≥7.5 mg of glucocorticoids (n = 2045, 9.4%) had a HR of 2.33 (95% CI 1.68, 3.22) of incident diabetes compared with patients who were not prescribed oral glucocorticoids in multivariate models (n = 14338, 65.8%, Table 4). In a sensitivity analysis with additional adjustment for CDAI, glucocorticoids dosage was still associated with diabetes development. No statistically significant increase in risk of diabetes was found for overweight RA patients, although the point estimate of the model (HR 1.91, 95% CI 0.82 4.42) trended towards an increased risk. Obese RA patients, defined by a BMI value >30 kg/m2, had a steep increase in diabetes risk (HR 6.27, 95% CI 2.97, 13.25, Table 4). If assessing the effect of glucocorticoid dose of ≥7.5 mg in patients with a BMI >30 kg/m2, the diabetes risk (HR 2.6, p-value 0.01, 95% CI 1.27 5.47)) was comparable to what was observed for the overall group of patients on glucocorticoid doses of ≥7.5 mg.

**Table 4:** Cox regression models assessing the relationship between prescription of glucocorticoids at index visit and incident diabetes (top rows) and BMI at index date and incident diabetes (bottom rows), adjusted for DMARD use, age and family history of diabetes.

	Hazard ratio	95% CI	P-value
***Glucocorticoid prescription at index visit***			
*0 mg*	*Reference*		
*1–2*.*5 mg*	1.35	0.89, 2.03	0.6
*3–7 mg*	1.95	1.47, 2.58	<0.01
*≥ 7*.*5 mg*	2.33	1.68, 3.22	0.02
***BMI***			
*< 25*	*Reference*		
*25–30*	1.91	0.82, 4.42	0.13
*> 30*	6.27	2.97, 13.25	<0.01

## Discussion

In this study, a significantly reduced risk of diabetes was found in RA patients treated with TNF inhibitors when adjusting for covariates such as disease activity and BMI. In addition, the risk of diabetes increased with increasing doses of glucocorticoids. The results might have clinical implications when choosing RA treatment in patients with an increased risk of diabetes.

RA patients have a known increased risk of cardiovascular disease, which add to both morbidity and mortality in these patients [8, 27, 28]. The combined effects of RA and diabetes on cardiovascular risk are not well understood, but an interaction between these two risk factors is likely. It is thus of special interest to prevent diabetes development in these patients. The lower hazard ratio for diabetes observed in patients receiving TNF inhibitors in our study underline that choice of RA treatment can have broad implications for the patient. However, the effects observed may be a result of modulation of disease activity by bDMARDs and not a direct effect of TNF inhibitor treatment. The trends observed for the other DMARD categories did not reach statistical significance, although this might have been due to lack of statistical power and should be interpreted within that context.

Treatment regimens with a combination of DMARDs and glucocorticoids have been shown to effectively inhibit joint damage in RA, and glucocorticoids are commonly used in RA treatment [2, 3, 29]. Contrary to the protective effect of TNF inhibitors, glucocorticoid use was associated with a significant increase in risk of diabetes in our data, especially in patients prescribed doses of 7.5 mg daily or more. Additionally, obese patients had a steep increase in risk of diabetes compared to RA patients with a BMI ≤25 (HR 6.27, 95% CI 2.97, 13.25). This risk is much higher than what has previously been reported in a Swedish study [13], and supports the importance of physical activity and dietary advice in this population. The higher rate might be due to differences in the obese populations (e.g. obese patients in the US having higher BMI and participating in less physical activity than obese patients in Sweden), in prescription patterns for glucocorticoids, or other unknown factors. Glucocorticoids are commonly prescribed for obese RA patients with insufficient control of disease activity. Further studies are needed to investigate potential interactions between the observed risks of diabetes development associated with obesity and glucocorticoids, the effects of cumulative glucocorticoid dosages, and whether alternative approaches such as intra-articular glucocorticoid injections should be preferred in these patients.

The study has certain limitations. Each reported case of incident diabetes was confirmed by a combination of chart reviews and patient interviews by the individual investigation sites, but HbA1c information was not collected. The case confirmation process might be the reason we observed a rather low incidence rate of diabetes compared to previous reports from high risk patients [30], however we have little reason to believe that this should be skewed between exposure groups. A delayed diagnosis of diabetes might potentially lead to a case being attributed to the wrong treatment episode. The treatment categories and choice of comparator group was predefined to allow for comparison with previous results. We chose to not make post-hoc adjustments although the data showed the comparator group, other non-biologic DMARDs, to be relatively small. An alternative comparator group would have been methotrexate treatment, but previous results indicating methotrexate to influence serum glucose levels [31] support a potential effect of methotrexate on diabetes development, making it less suitable as comparator in these analyses. For some of the analyses a dataset of only patients who were DMARD-naïve at inclusion would have been ideal, however this would be difficult to obtain due to the number of observations necessary, and would limit the opportunity to study biologic DMARDs. A comparator group of healthy individuals would have been of interest, but were not available in this cohort. The inclusion of known risk factors for diabetes, e.g., disease activity, family history and BMI, in the Corrona data collection is an advantage of the current study, allowing us to better control for confounding by indication in analyses of DMARD and glucocorticoid exposure. Additional strengths include the large number of observed treatment regimens, and the diversity of the rheumatology centers participating in Corrona. Although it is difficult to fully adjust for differences between the treatment groups in an observational study, the results of the study are strengthened by the comparability to results found in previous studies RA and diabetes are two chronic conditions linked through a multitude of factors, including the effects of anti-inflammatory treatment used to abate disease activity in RA. The current study supports that potent treatment of RA with bDMARDs correlate with a reduced risk of diabetes, thus reducing the burden of disease and risk of cardiovascular disease in these patients. Contrary, an increased risk of diabetes was found in patients prescribed glucocorticoid doses of 3 mg daily or more. RA treatment in patients with a known increased risk of diabetes, such as BMI over 30 kg/m2, should be monitored with this in mind.
